# Barriers and facilitators to the implementation of virtual reality as a pain management intervention in outpatient physiotherapy practices: a qualitative analysis

**DOI:** 10.1186/s12913-026-14967-4

**Published:** 2026-06-16

**Authors:** Alexander Elser, Christian Kopkow, Axel Schäfer

**Affiliations:** 1 Faculty of Social Work and Health, University of Applied Sciences and Arts Hildesheim/Holzminden/Göttingen, Hildesheim, Germany; 2 Faculty 4 for Human Sciences, Department of Physiotherapy, Brandenburg University of Technology Cottbus - Senftenberg, Cottbus, Germany

**Keywords:** Virtual reality, VR, Chronic pain, Physiotherapy, Implementation, Barriers, Facilitators, Qualitative research, Interviews

## Abstract

**Background:**

Chronic pain is a leading global cause of disability. There is evidence supporting the efficacy of virtual reality (VR) interventions for improving pain and function in patients with chronic pain. However, use of VR in physiotherapy practice remains limited. This study examined the pre-implementation barriers and facilitators experienced by physiotherapists working in Germany when implementing VR for chronic pain management in outpatient settings.

**Methods:**

Physiotherapists participating in a VR implementation study were interviewed using semi-structured interviews. The interviews were transcribed and analyzed using qualitative content analysis. The identified barriers and facilitators were categorized into domains of the Theoretical Domains Framework.

**Results:**

Based on the interviews with nine physiotherapists, the pre-implementation key barriers included environmental barriers, such as time limitations and lack of insurance reimbursement, knowledge barriers in relation to chronic pain management and VR content, professional role barriers, such as VR being perceived as outside the scope of physiotherapy and decision-making barriers, such as patient selection. The primary facilitators were environmental opportunities, such as VR being a unique asset of the practice or a dedicated area for VR therapy. Additional facilitators included positive expectations for the rehabilitation process and the belief that VR is an opportunity of growth for physiotherapy as a profession.

**Conclusions:**

Physiotherapists recognize the therapeutic potential of VR, but anticipate significant implementation challenges related to environmental restrictions, knowledge gaps, and professional role conflicts. However, they also identified potential facilitators, such as VR’s unique assets for practices and its benefits for patient empowerment and professional advancement. Successful adoption requires multifaceted strategies that address reimbursement policies, provide enhanced training in areas such as pain neuroscience and VR applications, and facilitate workflow integration. Future research should validate these findings across diverse healthcare systems to support the integration of VR in chronic pain care.

**Trial registration:**

The study was registered with the German Clinical Trials Register on April 14, 2023 (ID: DRKS00030862).

**Supplementary Information:**

The online version contains supplementary material available at 10.1186/s12913-026-14967-4.

## Background

Pain-related conditions account for 22.2% of all years lived with disability worldwide, with low back pain being the largest contributor [1]. In addition, the ageing of the global population and the resulting growing burden of non-communicable diseases, such as low back pain and other chronic pain conditions, pose significant challenges to healthcare systems worldwide [2].

A biopsychosocial approach to the management of low back pain and chronic pain conditions is recommended [3–6]. The most effective non-pharmacological treatments options are physical activity, exercise [3–7] and psychological approaches, such as cognitive behavioral therapy In outpatient healthcare settings, healthcare professionals such as physiotherapists commonly carry out treatments and recognize the importance of biopsychosocial musculoskeletal pain management, especially for non-specific chronic low back pain [8, 9]. However, integrating psychological aspects is challenging due to the discrepancy between patients’ beliefs about pain and evidence based therapy principles [10]. Additionally, physiotherapists report feeling inadequately trained, having difficulty interpreting severe pain when objective findings are limited and lacking confidence in addressing psychosocial factors [8, 11, 12].

Digital health interventions (DHI) such as mobile apps, web-based programs, or Virtual Reality (VR) applications, can support healthcare professionals in the biopsychosocial management of people living with chronic pain. A meta-analysis showed improvement in pain-related symptoms such as pain intensity (Standardized Mean Difference [SMD] 0.25 95% CI: 0.03, 0.46), depression (SMD 0.30 95% CI: 0.17, 0.43) and anxiety symptoms (0.37 95% CI: 0.05, 0.69) [13]. Improving patient autonomy, promoting self-management and reducing pharmacological interventions are additional beneficial effects reported [14]. However, more research is needed to understand the underlying mechanisms of the psychological effects and economic benefits of DHIs, which is essential to develop effective DHIs to support chronic pain management [14].

One promising DHI are therapeutic VR interventions, which target the biopsychosocial model through distinct working mechanisms. The biological mechanisms of VR include inducing neuroplastic changes and improving motor function [15]. Psychologically, VR can facilitate distraction from pain processing, cognitive restructuring, and emotional regulation [15]. Socially, it can foster the empowerment of patients [16]. Combination approaches integrate elements to address multiple biopsychosocial domains. VR has shown positive effects in acute and chronic pain conditions, such as pain relief during medical procedures in adults (SMD 0.78, 95% CI: 1.00, 0.57) [17] and children [18], cancer patients (SMD, − 0.88, 95% CI: −1.15, − 0.60) [19] and chronic pain management [20]. However, only 2.7% of physiotherapists working in Germany reported having used VR for the treatment of people living with chronic musculoskeletal pain in 2024 [21], in the Netherlands 7% of Physiotherapists used VR [22].

One possible reason for this is the lack of systematic and targeted strategies for the implementation of VR interventions in the treatment of people with chronic pain in physiotherapy. In order to develop a targeted implementation strategy, it is mandatory to classify barriers and facilitators using theoretical frameworks, such as the Theoretical Domains Framework (TDF) [23, 24]. The TDF was created to improve healthcare researchers’ access to psychological theory by providing a systematic and simplified approach to behavior change theories. With 14 theoretical domains derived from 33 theories and 128 constructs, this framework serves as a valuable tool for identifying and categorizing barriers and facilitators that influence professional behavior change in implementation processes. Based on these factors, a strategy can be developed using the Behavior Change Wheel (BCW) [25]. The BCW is a comprehensive framework that incorporates behavioral theory to effectively capture and address the mechanisms of action within implementation interventions. Developed through expert consensus and a rigorous validation process, the wheel is organized into three levels. The central element is the COM-B model, a behavior change framework that includes aspects of capability (both physical and psychological), opportunity (both social and physical), and motivation (both automatic and reflective). It is proposed that people need these three factors to increase the likelihood of performing the behavior in question [26].

Already known barriers and facilitators to the implementation of VR in physiotherapy can be used to develop an implementation strategy. Using the COM-B model, these barriers can be categorized as follows: In the capability category are technical limitations of VR devices, lack of tutorials/protocols and patients’ low gaming skills [27, 28]. In the opportunity category: Time constraints and lack of reimbursement [29, 30]. In the motivation category: Professional role conflicts (perceiving VR as outside the scope of physiotherapy) and positive expectations [27–30]. Healthcare professionals themselves can act as facilitators, influencing patients’ attitudes towards VR and reducing their fear of new technologies [27–30]. Patients also have positive expectations, describing VR as a fun and enjoyable treatment. However, these barriers and facilitators have been identified almost exclusively from the patient perspective [27]. In contrast, this study focuses on physiotherapists without prior clinical VR experience and explores their perceptions before implementation [31].

## Methods

### Study design

This is a qualitative study using semi-structured interviews. Adopting a constructivist paradigm, the study aimed to explore physiotherapists’ subjective experiences and perceptions of the barriers to and facilitators of implementing VR interventions. The TDF [23, 24] and the BCW [25] guided the data collection and analysis.

Ethical approval for the study was obtained from the Ethics Committee of the University of Applied Sciences and Arts Hildesheim/Holzminden/Göttingen, Germany, on 3 April 2023. The study was conducted in accordance with the Declaration of Helsinki. The reporting of this study follows the Standards for Reporting Qualitative Research (SQRQ) [32]. The study was registered with the German Clinical Trials Register (ID: DRKS00030862).

### Study context

This qualitative study is part of an implementation project that aims to implement a VR-based psycho-educational intervention (VRPI) for people living with chronic pain in outpatient physiotherapy settings. Prior to implementation of the VRPI, interviews were conducted with physiotherapists to develop a targeted implementation strategy. The strategy will then be carried out and evaluated as part of the implementation project. A separate publication provides an overview of the entire process [33]. The implementation project involves five physiotherapy practices (outpatient care) in Lower Saxony, Germany. Two weeks before the interviews, two therapists each were trained in the use of the VR device (Pico 4, ByteDance, 2022) and the VRPI and then invited to test them at home. The VRPI, called Reducept (Reducept BV, Version 1.6, 2024) is a psychological and educational intervention for the treatment of chronic pain. Reducept is designed to teach patients that pain can be influenced and managed by changing the way they think about their pain. In the game, the user navigates through the body in a spaceship and along the way, they learn about the mechanisms of pain. Reducept is based on the “Explain Pain” guidelines [34], which state that by understanding pain and influencing the cognitive, emotional and behavioral processes associated with it, patients will be able to reduce their experience of pain. In addition to pain education, the user plays several serious games that stimulate the visual, auditory, and proprioceptive systems to control chronic pain through distraction and relaxation. The VRPI uses Cognitive Behavior Therapies (CBT), Acceptance and Commitment Therapies (ACT), mindfulness and hypnotherapy techniques.

The training and interviews were conducted by one author (AE), a physiotherapist (MSc). Before and partly during his academic training, he gained 3 years of professional experience in outpatient physiotherapy practices. He was not known to the participants of this research prior to undertaking the study. While it was useful to have ideas, based on his own knowledge, about domains where barriers and facilitators might arise when implementing a VRPI in outpatient physiotherapy practices, he made a conscious effort as a researcher not to include these assumptions in the interviews and analysis.

### Sampling and data collection

Purposive sampling was used to recruit participants for the semi-structured interviews. Physical therapists who are part of the implementation project and who have attended the training courses on VR devices and the VRPI were approached in order to obtain the widest possible range of views on implementation and barriers and facilitators. One of the inclusion criteria was that they were employed in physiotherapy practices and were working at least 20 h per week. An individual 90-minute interview was then arranged with each of them. The interviews were conducted via online via Zoom (Zoom Video Communications, USA) and the audio was recorded.

For the semi-structured interviews, a guideline based on the TDF was used (Appendix 1). The guideline consisted of four parts. (1) Obtaining informed consent. (2) Providing an introduction to the procedure and content of the interview. (3) Guiding questions and possible follow-up questions. (4) Closing the interview by collecting demographic data, thanking the interviewee. The guiding questions were developed by first creating an open-ended question for each TDF domain to ensure that all relevant issues were addressed. Then, these questions were adapted to focus on physiotherapist behaviors that needed to change for the VRPI to be implemented. The guideline was pre-tested with two physiotherapists, who were comparable to the sample and who were not involved in the study.

### Data analysis

The audio data was transcribed using the F4x program and then edited by one author (AE) according to Kuckartz’s transcription rules. These rules provide a standardized framework for verbatim transcription of qualitative interviews, including documentation of non-verbal expressions, pauses and emphasis, in order to preserve the original context [35]. The transcripts were then analyzed according to Kuckartz’s qualitative content analysis [36]. Content analysis is a systematic, rule-guided method of structuring and interpreting textual data through inductive and deductive category development. The fourteen TDF domains were used as deductive main categories. The coding segments found were first divided into barriers and facilitators, and then thematic subcategories were developed for each. Two of the authors (CK and AS) independently checked the assignment of the coded segments to the main topics, the classification into barriers and facilitators, and the development of the subcategories.

## Results

### Participants

Nine physiotherapists (3 female; 6 male) participated in the semi-structured interviews. The mean age was 32.44 years, the mean years of professional experience was 8.11 years, and the mean number of chronic pain patients seen per week was 7.33. Participant characteristics are summarized in Table 1.

**Table 1:** Participant characteristics

Participants (*n*)	9
Mean age in years (SD, range)	32.44 (11.30, 24–53)
Gender Female Male	3 6
Highest degree Vocational certificate Bachelor´s	6 3
Mean professional experience in years (SD, range)	8.11 (7.74, 2–24)
Patient´s per week with chronic pain conditions (SD, range)	7.33 (5.92, 1–20)

SD = Standard deviation

### Themes

After categorizing the coded segments into barriers and facilitators, barriers to the use of the VRPI in outpatient physiotherapy practices could be assigned to eight TDF domains (Table 2). Subthemes were developed based on the content of the themes through an inductive analysis of the interview data. Then, the frequency of mentions was used as a supporting criterion to confirm the relevance of these themes, assist in their organization and prioritization, and determine their substantive significance within the TDF framework. This approach ensured that the final categorization was driven by thematic meaning, with frequency serving as an additional layer of validation. One or more subthemes were identified for each of the TDF domains. All subthemes can be found in Appendix 2. The frequencies in the subthemes do not always match the frequency for the TDF domain. This is because not all coded segments could be assigned to subthemes, for example because the statement was too general.

**Table 2:** Barriers to using the VR-based psycho-educational intervention

TDF Domain (Frequency)	Subthemes (Frequency)
Environmental context and resources [30]	- Not enough time to implement in therapy [11] - No prescription/no payment for VR therapy [9] - Patients may not be interested [7] - Blocking of therapy rooms [3]
Knowledge [16]	- Lack of knowledge regarding the application of psychological and educational approaches when treating people with chronic pain [9] - Lack of knowledge about the VRPI [6]
Social/professional role and identity [14]	- VR therapy is not a part of physiotherapy [10] - VR therapy could eliminate the need for physiotherapy [2] - VR therapy steals time from other therapies [1]
Memory, attention and decision processes [14]	- Uncertain decision criteria for or against the use of the VRPI [12]
Skills [9]	- Uncertain about how to navigate the VR devices [6] - Lack of skills and assessment to verify patient affinity for technology [2]
Emotion [5]	- Triggers fear, anger, excessive demands in patients [3]
Belief about consequences [3]	- Overstraining patients with VR devices can lead to negative emotions [2] - VR devices could scare patients [1]
Beliefs about capabilities [1]	- As a physiotherapist, you have no influence on chronic pain anyway [1]

TDF = Theoretical Domains Framework; VR = Virtual Reality, VRPI = VR-based psycho-educational intervention

The most frequently coded barriers were assigned to the TDF domain “Environmental Context and Resources” (30 statements). Physiotherapists frequently mentioned that there was not enough time in their working day to implement and carry out a VRPI (*n* = 11).

And the time factor. I don’t know how long a session actually lasts, but theoretically a patient only has about 20 min. And just setting up all the technology, switching it on, then really getting into the training, dismantling it again, leaving the room. It’s hard to imagine that it all works in 20 min. (Phase2\Interview_03:64)

Another subcategory showed that therapists mentioned the fact that VR therapy is not yet covered by health insurance in Germany as a barrier to implementation [9].

The second most frequently coded barriers were in the domain Knowledge [16]. Within this domain, it was found that physiotherapists lack of knowledge regarding the application of psychological and educational approaches when treating people with chronic pain [9].

Mirror therapy is the first thing that comes to mind, although I’m more familiar with it in the area of phantom pain. But I also know that it is also used for chronic pain. Hm, yeah, that’s actually the only thing I can think of, really. (Phase2\Interview_02:10)

The Memory, Attention, and Decision Processes [14] domain revealed practical uncertainties, including unclear patient selection criteria and ambivalence about applying the VRPI [12].

Another important barrier was the Social/Professional Role and Identity domain [14]. Physiotherapists tend not to consider VR interventions as part of physiotherapy [10].

I don’t think I would have categorized VR interventions as physiotherapy per se, but rather as something that I think could be used well in psychotherapy as an accompaniment. (Phase2\Interview_01:22)

Facilitators were identified in 10 of the 14 TDF domains (Table 3). As with the barriers, the frequencies in the subthemes do not always match the frequency for the TDF domain. All subthemes can be found in Appendix 2.

**Table 3:** Facilitators to using the VR-based psycho-educational intervention

TDF Domain (Frequency)	Subthemes (Frequency)
Environmental context and resources [21]	- VR therapy as a unique selling point for physiotherapy practices [6] - Creation of a permanent space for equipment and VR therapy [5] - Using the screencast function of the VR device [3] - VR therapy as a prescription [3]
Belief about consequences [19]	- Better visualization of the education in the therapy of people living with chronic pain [7] - A way to learn about their condition [5] - Empower through VRPIs [4] - Increasing the motivation [2] - Reduction of therapist work load [2] - Distraction from pain [1]
Social/professional role and identity [9]	- Opportunity for advancement in physiotherapy [4] - Successfully using/implementing the VRPI can help to strengthen the work as a therapist [3]
Intentions [6]	- Intention to use VRPI early and sustainably in the therapy [3]
Social influences [6]	- Inquiries from patients or colleagues about VR therapy [3] - Knowledge Broker in practice [3]
Skills [5]	- Time to test and use the VRPI in a real-world setting [2] - Instruction manual + step-by-step protocol for selecting the VRPI in the VR device [2]
Memory, attention and decision processes [5]	- Good visibility of the VR device in practice [3] - Promotion of VRPI in practice [1]
Optimism [2]	- Positive basic attitude towards VR therapy [2]
Beliefs about capabilities [1]	- Positive experiences with the use of VR [1]
Emotion [1]	- Pride when the integration is working and the positive benefits to the people living with chronic pain are evident [1]

TDF = Theoretical Domains Framework; VR = Virtual Reality, VRPI = VR-based psycho-educational intervention

The most frequently coded facilitators were in the TDF domain Environmental Context and Resources (21 statements). Within this domain, physiotherapists often mentioned that therapeutic VR can be a unique selling point for physiotherapy practices and thus a facilitator in implementation processes (*n* = 6). They also see the creation of a permanent space where therapeutic VR is used as a facilitator in practices [5].

So of course, it would be ideal if you had a room with VR use only and the glasses were somehow hanging there, for example, which means they don’t have to be packed and unpacked and tidied up and I don’t know, they’re just always connected and hanging there. So, the WLAN and so on and the space is right, so that the patient also has the feeling, okay, here is a room where I have enough space and nothing happens. Nobody comes in, you’re undisturbed. I think that would be ideal. (Phase2\Interview_02:64)

The second most frequently mentioned facilitators were coded in the domain of beliefs about consequences [19]. This reflects physiotherapists’ high expectations of therapeutic VR in the treatment of people living with chronic pain. These include the belief that therapeutic VR improves pain education [7], enabling people to learn more about their condition [5] and empowering them to become active participants in their treatment [4].

Getting to grips with the pain and understanding that relaxation, boundaries and all that sort of thing also play a part. So that the mind is also a big factor when it comes to pain. That is important to me. So that’s what I think is important for patients to take away with them, because I think it gives them a lot of input that they can use.(Phase2\Interview_02:31)

Social/professional role issues may also facilitate the implementation of therapeutic VR in physiotherapy practices [9]. In particular, the possibility of advancing the profession of physiotherapy [4] and strengthening one’s own work as a physiotherapist [3].

## Discussion

The aim of this study was to explore pre-implementation barriers and facilitators to the implementation of a VRPI in the care of people with chronic pain among physiotherapists working in Germany, in order to develop an implementation strategy. Influencing factors in 10 domains were identified using the TDF. In the domain of environmental context and resources, the main barriers are a lack of time and the fact that German health insurance does not cover VR therapy. In the knowledge domain, therapists often lack an understanding of chronic pain and VRPIs. Uncertainty about when and how to use VR is evident in the memory, attention, and decision processes domain. Furthermore, many physiotherapists do not consider VR to be part of their profession, creating challenges regarding their professional role and identity.

The most often mentioned facilitator in the environmental context and resources domain is the perception of VR as a unique selling point that could provide a competitive advantage to practices. The availability of a specific space in the practices for VR interventions is also beneficial. In the beliefs about consequences domain, physiotherapists have high expectations of VR interventions, believing that it will improve the visualization of chronic pain education, increase patient knowledge, and motivate active participation in treatment. In the professional role domain, facilitators support the adoption of VR interventions by advancing the physiotherapy profession and empowering practitioners. These facilitators highlight the potential of VR to improve patient care and encourage innovation in physiotherapy practice.

Several barriers identified in this study to the implementation of VRPIs in chronic pain care were also found in various healthcare settings. These barriers include insufficient time and reimbursement for VR interventions [29, 30]. As in other areas of healthcare [30] and physiotherapy [28], the lack of recommendations and standardization of how, when and for whom VR interventions should be used is also a barrier. This study indicates that when healthcare professionals are inexperienced with a condition’s treatment options, it can hinder the implementation of digital interventions involving those options. Another new finding is that, when implementing digital interventions, it is important to consider whether the physiotherapists view the interventions as part of their professional scope. Currently, physiotherapists primarily define themselves in terms of their clinical competencies [37]. However, surveys in Norway, Australia, Ireland, and Germany show that a significant number of physical therapists use or would use digital technologies [38–41]. Therefore, a key aspect of implementing VR and other digital technologies is involving professionals in the design and introduction of digital interventions through participatory development processes at an early stage. This increases their sense of professional ownership of the intervention and promotes acceptance [42].

Regarding the identified facilitators for the implementation of a VRPI in the management of people with chronic pain, it is evident that already known facilitators, such as a high expectation of therapeutic success of VR, are also present among the interviewed physiotherapists regarding the VRPI [28]. These outcomes may differ among physiotherapists with more expertise in treatment of people with chronic pain as their advanced training might influence their perception of VR’s therapeutic value. In addition, physiotherapists see VR as an opportunity to further develop the profession and strengthen their own work, which stays in contrast to the earlier mentioned barrier related to their professional role. This underlines the importance of the intrinsic values of the people involved in implementations playing a central role in the success of implementation processes [43]. Therefore, implementation should be an integrated process rather than a separate phase, with co-creation involving stakeholders from the design stage onward.

Given the numerous barriers and facilitators identified, it is essential to use systematic strategies when implementing VRPIs in the healthcare setting. These can be developed from the local identified barriers and facilitators. Determinate and process frameworks, such as the TDF [23] combined with the BCW [25] or the NASSS Framework [44], are helpful and have been used by others. Some research has already identified evidence-based implementation strategies that have worked in other studies. During the pre-implementation phase, strategies like educational activities, such as meetings and informational materials, as well as activities, such as identifying and preparing a knowledge broker had impact on the implementation outcomes [45]. Implementation strategies that impacted the outcomes included providing coaching and support, engaging additional key partners, participating in quality improvement activities, and developing and implementing a quality monitoring tool [45]. The barriers identified in this study in the domains of environmental context and resources, knowledge, and professional role and identity suggest potential implementation strategies. These strategies may include environmental restructuring, education, and the introduction of a knowledge broker.

## Limitations

This is one of the first studies to explore the barriers and facilitators of implementing VRPIs for people with chronic pain from the perspective of physiotherapists. One limitation is that only a small number of physiotherapists were able to participate since study participants were recruited from cooperating practices. Additionally, the identified barriers and facilitators, particularly those related to limited knowledge of the VRPI and the psychological and educational treatment of people with chronic pain, may not be applicable to other contexts.

## Conclusions

This qualitative study identified barriers and facilitators anticipated by physiotherapists regarding the implementation of a VRPI for managing chronic pain in outpatient care. The findings highlight significant challenges in the domains of environmental context, resources, knowledge, and professional identity. However, they also reveal notable opportunities, such as professional advancement and improved patient engagement. Addressing these barriers through targeted education, structural adaptations, and systematic implementation strategies may foster the sustainable integration of VR interventions in physiotherapy practice. Future research should explore these approaches in broader and more varied settings to enhance the generalizability and impact of implementation efforts.

## Supplementary Information

Below is the link to the electronic supplementary material.

Supplementary Material 1

Supplementary Material 2

## Data Availability

The datasets generated and/or analyzed during the current study are not publicly available due to privacy considerations but are available from the corresponding author on reasonable request.

## Abbreviations

ACT: Acceptance and Commitment Therapy
AIM: Acceptability of Intervention Measure
BCW: Behavior Change Wheel
CBT: Cognitive Behavioral Therapy
COM-B: Capability, Opportunity, Motivation—Behavior
DALY(s): Disability-Adjusted Life-Years
DHI(s): Digital Health Intervention(s)
DRKS: Deutsches Register Klinischer Studien (German Clinical Trials Register)
EBP(s): Evidence-Based Practice(s)
FIM: Feasibility of Intervention Measure
HALE: Healthy Life Expectancy
IAM: Intervention Appropriateness Measure
NASSS: Non-Adoption, Abandonment, Scale-up, Spread, and Sustainability
SD: Standard Deviation
SQRQ: Standards for Reporting Qualitative Research
TDF: Theoretical Domains Framework
VR: Virtual Reality
YLD(s): Years Lived with Disability
