# Supplementary material for: Barriers and facilitators to the implementation of virtual reality as a pain management intervention in outpatient physiotherapy practices: a qualitative analysis
Source: BMC Health Serv Res. 2026 Jun 16;26:842. doi: 10.1186/s12913-026-14967-4 (PMC13277262; doi:10.1186/s12913-026-14967-4)
Supplement: Supplementary file 1 — Supplementary Material 1 [file 12913_2026_14967_MOESM1_ESM.pdf]

# I. Before the interview

## 1. Informed consent

The document must be sent in advance by email and returned after signing.

# II. Introduction

## 1. Introduction

This interview is about the practical implementation of virtual reality intervention. It is important to me to mention that there are no right or wrong answers. All of your experiences and thoughts on VR intervention and other topics are valid and important, as they provide valuable insights into the feasibility of VR intervention and, ultimately, into improving physiotherapy care for people with chronic pain.

In my guide, I have listed a number of topics, and I will ask you a question about each one. You can then tell me everything that comes to mind or seems important to you in relation to that question. I will give you time to think, so there will be pauses throughout the interview during which you can consider whether you have said everything that is important to you. I will not interrupt you or comment on anything in order to avoid disturbing or influencing your thoughts as much as possible. However, I may ask you a question at one point or another, for example because I am not sure whether I have understood what you have said correctly or because I would like to hear more about a particular aspect.

To do this, I will occasionally take notes while you are speaking. So if I lower my head from time to time during the conversation, I am looking at my guide and my notes. Please do not let this distract you; I am still listening to you with interest. The interview will take about an hour.

The plan is to use the results of the interview to develop and implement a strategy for introducing VR intervention into your physical therapy practice. This process will be anonymous, meaning that names, locations, and other information will be anonymized so that no conclusions can be drawn about you or your practice.

## 2. Questions

Do you have any questions about the interview process or the evaluation of the data?

Then I'll start recording now and we'll begin the interview.

## 3. Start recording

## III. Guide

- Please describe your typical patients with chronic pain who come to you for treatment. (Knowledge)
  - What do you understand when you hear that a patient has chronic pain?

- What therapies do you know of for patients with chronic pain? (Knowledge)
  - Where did you learn about these therapies?

- What thought process guides you when deciding how to treat your patients with chronic pain? (Memory, attention, decision-making processes)
  - In which situations would you decide to treat your patient with a VR intervention? In which situations would you decide against it?
  - What would help you remember the VR intervention?
  - What would help you decide in favor of the VR intervention?

- What personal goals have you set for yourself in terms of how you treat patients with chronic pain? (Goals)
  - What goal have you set for yourself in terms of VR intervention?

- What skills do you think are needed to use VR intervention in everyday life? (Skills/abilities)
  - To what extent do you think you have these skills?
  - How could you develop these skills?

- To what extent do you believe that the use of VR interventions is part of your work as a physical therapist? (Professional role)
  - How is the use of this VR intervention compatible with your expertise, or does it contradict it?

- How determined are you to use VR intervention in your treatment of patients with chronic pain? (Intention)
  - What do you intend to achieve by using VR intervention?

- What do you think will happen if you use VR intervention? (Expected consequences)
  - What advantages or disadvantages do you think this would have for patients?
  - What positive or negative consequences would this have for you?
  - How do you assess the relationship between the potential benefits and the costs or effort involved in VR intervention?
  - What advantages or disadvantages could arise for your practice?

- In your opinion, what would help you and your colleagues to use VR interventions regularly with patients suffering from chronic pain? (Behavioral regulation)

- Do you or your practice have ways of estimating how often you have performed a VR intervention? If so, what are they?
- What might these look like?

- To what extent could your workplace influence the use of VR intervention for patients with chronic pain? (Environmental factors and influences)
  - Which organizational factors (e.g., time factors) could make it easier or more difficult for you to integrate VR intervention into your everyday clinical practice?
  - To what extent would something in your work environment need to be changed so that you could implement VR intervention?
  - To what extent does the equipment at your workplace (e.g., materials, rooms) correspond to what you need for therapy with VR intervention?
  - What legal framework conditions could influence the implementation of VR intervention?

- Let's move on to VR glasses as a technical device: To what extent could VR glasses and their functions influence the use of VR intervention in patients with chronic pain? (Environmental factors and influences)

- How easy or difficult do you think it will be for you to treat patients with chronic pain using this VR intervention? (Self-efficacy)
  - What would you be particularly good at?
  - What would you find difficult?
  - What could help to (further) boost your confidence?

- What influence could other people (e.g., doctors, colleagues from other healthcare professions, patients, relatives) have on whether or how you integrate the VR intervention into your everyday clinical practice? (Social influences)
  - What is your colleagues' attitude toward integrating the VR intervention?
  - What role does your employer play in integrating the VR intervention?
  - Do you feel that the VR intervention meets your patients' expectations of treatment?

**From here on optional (if there is still time and if not already mentioned):**

- What emotional response might treating patients with chronic pain using VR intervention, or even thinking about it, trigger in you? (Emotion)
  - How do these feelings influence your choice of treatment methods?
  - Do the emotions or psychological distress of patients with chronic pain influence your decision on how to treat them?
- How confident are you that the implementation of VR intervention in practice will be successful? (Optimism)
- How could clearly defined goals or rewards help you to use VR intervention regularly? What might these look like? (Reinforcements and goals)

**Final question**

- What might your therapy with patients suffering from chronic pain look like in 10 years? (Memory, attention, decision-making processes)
  - How do you imagine VR interventions being used in the future therapy of patients suffering from chronic pain?
  - Do you think VR could become an important therapeutic method? Why?

## IV. After the interview

1. Request for demographic data
2. Note regarding the sending of the transcript
3. Acknowledgment
4. Farewell
