# Supplementary material for: Barriers and facilitators to the implementation of virtual reality as a pain management intervention in outpatient physiotherapy practices: a qualitative analysis
Source: BMC Health Serv Res. 2026 Jun 16;26:842. doi: 10.1186/s12913-026-14967-4 (PMC13277262; doi:10.1186/s12913-026-14967-4)
Supplement: Supplementary file 2 — Supplementary Material 2 [file 12913_2026_14967_MOESM2_ESM.pdf]

Barriers to using the VR intervention

TDF Domain (Frequency)	Subthemes (Frequency)	Belief statements (interview number; paragraph) [translated into English]
Environmental Context and Resources (30)	- Not enough time to implement in therapy (11)	- And the time factor. I don't know how long a session actually lasts, but theoretically a patient only has about 20 minutes. And just setting up all the technology, switching it on, then really getting into the training, dismantling it again, leaving the room. It's hard to imagine that it all works in 20 minutes. (Phase2\Interview_03:64)
	- No prescription/no payment for VR therapy (9)	- Yes, well, I think it could be a problem on the one hand because VR itself, here in Germany at least, is not yet approved as a recognized therapy method, i.e. it is not covered by health insurance companies because VR is not yet recommended consistently. (Phase2\Interview_03:68)
	- Patients may not be interested (7)	- I could imagine that there are some people who, because this whole VR topic is something that is not yet so widespread, might feel that it is not being taken seriously, that they think okay, this is somehow here, I'm going to physiotherapy and I actually expect something different than being put on glasses where I'm somehow, I don't know, driving around in a spaceship and have to fulfill some tasks, that this might be something like that. (Phase2\Interview_08:68)
	- Blocking of therapy rooms (3)	- You have to look at the rooms. So if you occupy a room that might be needed for something else. (Phase2\Interview_07:46)
Knowledge (16)	- - Lack of knowledge regarding the application of psychological and educational approaches when treating people with chronic pain (9)	- Mirror therapy is the first thing that comes to mind, although I'm more familiar with it in the area of phantom pain. But I also know that it is also used for chronic pain. Hm, yeah, that's actually the only thing I can think of, really. (Phase2\Interview_02:10)
	- Lack of knowledge about the VRPI (6)	- And that's how I can imagine it working with Reducept. I can well imagine that it works. But it's just not really clear to me yet. How, why, and wherefore? (Phase2\Interview_06:18)

Social/Professional Role and Identity (14)	- VR therapy is not a part of physiotherapy (10)	- I don't think I would have categorized VR interventions as physiotherapy per se, but rather as something that I think could be used well in psychotherapy as an accompaniment. (Phase2\Interview_01:22)
	- VR therapy could eliminate the need for physiotherapy (2)	- Well, of course I'm giving up a bit of my work. So I don't want people to say at some point that physio is superfluous if we only have VR. I can see that as a disadvantage. Or that patients then say that I don't need all that anymore. I have my VR glasses here now. (Phase2\Interview_03: 56)
	- VR therapy steals time from other therapies (1)	- So I still see a bit of a difficulty in establishing this and that the instruction etc. then comes at the expense of treatment time. And I find that difficult. (Phase2\Interview_02:56)
Memory, Attention and Decision Processes (14)	- Uncertain decision criteria for or against the use of the VRPI (12)	- I think I'm a bit intuitive at first. Sure, you have the first intake, the anamnesis, and then of course a lot is about tactile contact. What is the person's state of tension? Is it the normal tone? Is it rather flaccid? Is he rather hypertonic, how does the whole thing feel and, of course, the visual findings. Is the person sitting in front of me very tense or is he sitting rather relaxed? Or is he a little slacker who can barely hold himself up on the chair? After that, I usually decide whether we should focus more on movement or whether I should work on lowering tone. (Phase2\Interview_06:12)
Skills (9)	- Uncertain about how to navigate the VR devices (6)	- So really going through the individual levels again and now simply handling the glasses themselves is not that difficult. It's more the menu navigation, how do I get where. So, I would just have to get used to it a bit more. (Phase2\Interview_06:58)
	- Lack of skills and assessment to verify patient affinity for technology (2)	- It would help me if there was a tool, maybe not, but somehow a way of finding out how tech-savvy a patient is. Of course, they don't have to be super tech-savvy to be able to use these glasses, but to what extent are they open? To be able to accept that. I still find that difficult to assess. And that there are somehow three, let's say, guiding questions by which you can perhaps recognize this a little. Is

		this a patient who is open to it and who perhaps also has the technical know-how or not? Exactly, so I think that would help me. (Phase2\Interview_02:21)
Emotion (5)	- Triggers fear, anger, excessive demands in patients (3)	- When you find yourself in such a different world for the first time and, let's say, are taken away from your normal impressions, it can certainly be frightening at first. I would say that it could initially be a loss of control rather than a gain in control. (Phase2\Interview_04:58)
Belief about Consequences (3)	- Overstraining patients with VR devices can lead to negative emotions (2)	- Possibly also anger, if you can't deal with it well now, then it would also be a question of whether they attribute it to themselves, that they can't do it now or something, these are perhaps also patterns that chronic pain patients can find themselves in, that they are always in this negative cycle. It's just that they can't do something. Yes, that could be confirmed if they have problems dealing with all the technical stuff. I could imagine that this could become a problem. (Phase2\Interview_04:82)
	- VR devices could scare patients (1)	- Yes, I could imagine that. Maybe if people are afraid of this. After all, if you wear it for a long time, it's heavy and relatively tight around the head, so patients are a bit scared because they no longer really have control over their environment. I could imagine that, because when you're in the program, you can only see the program. And yes, this entire visual surface is covered by the glasses, so that you can no longer see the space around you. And I could imagine that patients are put off by that because they give up a bit of control. (Phase2\Interview_03:46)
Beliefs about Capabilities (1)	- As a physiotherapist, you have no influence on chronic pain anyway (1)	- Because the experience I've had with chronic pain patients is that if they've already had so many treatments, I usually can't turn the tide myself. So you work a lot symptomatically and then there are phases where it gets better. Then you think you've got it now and then there are phases where you're back to square one. (Phase2\Interview_01:16)

TDF = Theoretical Domains Framework; VR = Virtual Reality, VRPI = VR-based psycho-educational intervention

Facilitators to using the VR intervention

TDF Domain (Frequency)	Subthemes (Frequency)	Belief statements (translated into English)
Environmental Context and Resources (21)	- VR therapy as a unique selling point for physiotherapy practices (6)	- It could, because it's very likely that not many practices have it yet, where it's a feature of the practice, so to speak, that you then also say yes, it's a lot about word of mouth, that you say, they're now using something new in the practice and it's helped me really well, why don't you go there too, so that the practice is recommended to others. (Phase2\Interview_01:38)
	- Creation of a permanent space for equipment and VR therapy (5)	- So of course, it would be ideal if you had a room with VR use only and the glasses were somehow hanging there, for example, which means they don't have to be packed and unpacked and tidied up and I don't know, they're just always connected and hanging there. So, the WLAN and so on and the space is right, so that the patient also has the feeling, okay, here is a room where I have enough space and nothing happens. Nobody comes in, you're undisturbed. I think that would be ideal. (Phase2\Interview_02:64)
	- Using the screencast function of the VR device (3)	- I mean, there is this function that allows you to see what the person in VR is seeing on an external device, so to speak. I think that would definitely be very useful if you somehow had a tablet next to it, for example, and could then simply see okay, that's what my patient is seeing right now, that's what's failing, and then guide him or her. I think that would definitely be something that would be very helpful in terms of technical support. (Phase2\Interview_08:66)
	- VR therapy as a prescription (3)	- So, then I would think that it could be prescribed as a kind of KG prescription. And perhaps not necessarily in this 20-minute period for someone who doesn't have private health insurance, but that you might also get an additional ten minutes like with a hot roll or fango (Phase2\Interview_07:76)

Belief about Consequences (19)	- Better visualization of the education in the therapy of people with chronic pain (7)	- The advantage is definitely this visualization, because I believe that it is difficult for many patients to imagine it. Even if you have pictures of the brain or the nervous system, I think it's still difficult for many people to understand it properly if they haven't had any training in it. And I think that's exactly what it's good for, to be able to visualize it properly and then perhaps get a feeling of control yourself when you shoot the pain signals, for example, or something like that. (Phase2\Interview_04:44)
	- A way for people with chronic pain to learn about their condition (5)	- Getting to grips with the pain and understanding that relaxation, boundaries and all that sort of thing also play a part. So that the mind is also a big factor when it comes to pain. That is important to me. So that's what I think is important for patients to take away with them, because I think it gives them a lot of input that they can use. (Phase2\Interview_02:31)
	- Empowering people with chronic pain through the VR intervention (4)	- But what I found really nice was that the patients were able to deal with the pain themselves in peace and quiet and that a lot was explained about their own pain. So I think it's nice that there are a lot of educational measures and that they can use them. So that they simply take action themselves. (Phase2\Interview_03:54)
	- Increasing the motivation of the people with chronic pain (2)	- Although now I've also noticed that VR glasses can perhaps also be a motivator for people who might not be so keen to sit down at a device and take part in active therapy, but that you can perhaps pick them up in this way. (Phase2\Interview_07:26)
	- Reduction of therapist work load (2)	- Advantage for me. It could be a relief. As the VR intervention takes a lot of the explanatory and advisory work off my hands, that is of course a relief. (Phase2\Interview_03:56)
	- Distraction from pain for people with chronic pain (1)	- It's also a distraction from the pain at first. So you're completely somewhere else. And I also don't believe that people concentrate on the pain at that moment. And that it could actually have a positive effect on the pain if they use this program. (Phase2\Interview_01:32)

Social/Professional Role and Identity (9)	- Opportunity for advancement in physiotherapy (4)	- I hope that patients will gain more insight into physiotherapy. I believe that physiotherapy is often still seen in a very niche way. They do this and this and this. And that the complexity of this profession, but also of the disease, is not yet in people's minds. So I believe that there might be more understanding of what we do and how comprehensive it actually is and everything that goes into it. Yes, I could imagine that this would simply change the way we view physiotherapy a little. What all our modes of action actually are, that it's not just: we do massage or we do active exercises, but it's also, yes, linked to mental health and perhaps also to the fact that I think it would be great, if it works, for patients to understand more again that they are responsible for themselves. (Phase2\Interview_02:52)
	- Successfully using/implementing the VRPI can help to strengthen the work as a therapist (3)	- Then another advantage. I could also imagine that patients could benefit from this in general. That's also an advantage for me because it brings therapeutic success and ultimately encourages me in my work. And in the end it's always the case when you realize, okay, you're successful with what you're doing, that it's more fun and more fulfilling. (Phase2\Interview_08:52)
Intentions (6)	- Intention to use VRPI early and sustainably in the therapy (3)	- Oh, it's already big. I would really like to try it out. Most patients are quite approachable and. Especially with pain patients, many are like that, as long as it helps. I'm open to anything. Do anything. (Phase2\Interview_03:73-74)
Social influences (6)	- Inquiries from patients or colleagues about VR therapy (3)	- I also believe that it's like that. Like a little running fire. So once you've done it with patients. So the patient, I think, also has an influence, if the patient says oh, I did something with VR glasses and it was totally cool and stuff, it might be demanded more by patients and is seen more as normal in the practice. But even if therapists talk about it more or offer it. It slips more into normality, (Phase2\Interview_02:78)

	- Knowledge Broker in practice (3)	- I could imagine that if, for example, a colleague says okay, I'll take over this area, I'm a bit responsible for it, that's my thing, these VR glasses, and he or she might regularly find out if there's anything new. Yes, and because there's always a bit of, yes, current information repeated from time to time and then maybe bring that into the team, if you have someone there, I could well imagine that that would definitely help. (Phase2\Interview_08:62)
Skills (5)	- Time to test and use the VR intervention in a real-world setting (2)	- Try it out here in practice, also with the rooms. How is it with adjusting the glasses or something like that? I haven't done that here in the rooms yet, but rather at home to get the final touches of security. (Phase2\Interview_05:22)
	- Instruction manual + step-by-step protocol for selecting the VRPI in the VR device (2)	- I think it's like a kind of protocol, so maybe it contains the most important things specifically for this app. So as far as the controls are concerned. Although I think it's actually relatively self-explanatory. That you have something there that you can look up again if you have any questions. (Phase2\Interview_08:32)
Memory, Attention and Decision Processes (5)	- Good visibility of the VR device in practice (3)	- Well, for me it's first and foremost the visibility, of course. I say, with VR goggles like these, you're in and out quickly. You can put them in a cupboard somewhere and of course they can be forgotten relatively quickly. For me, it's just really seeing things again and again and saying yes, exactly, there's still the method, we can fall back on it again (Phase2\Interview_06:46)
	- Promotion of VR intervention in practice (1)	- You could perhaps even put up a poster like this in the treatment room or somewhere. With VR glasses therapy for chronic pain or something, so that you can see it yourself, but maybe the patients can see it too. (Phase2\Interview_04:68)
Optimism (2)	- Positive basic attitude towards VR therapy (2)	- And I'm really looking forward to it and am rather positive about the whole thing. So I can already imagine that it will all have an effect and so I think it can start now and I also hope that it will bear fruit and really take hold somehow and be integrated into everyday

		physiotherapy. That would be nice, yes (Phase2\Interview_07:54)
Beliefs about Capabilities (1)	- Positive experiences with the use of VR (1)	- But I actually tested it on my parents, who are approaching 80, and simply put it on them. They were on amazingly quickly. I think it took about five minutes for my father. It took my mother maybe ten or 15 and then she was completely immersed. So that was exactly her thing. (Phase2\Interview_06:44)
Emotion (1)	- Pride when the integration is working and the positive benefits to the people with chronic pain are evident (1)	- If they make good use of it and take it with them, I think I would be very proud. Yes, I think I would like that. I would be really happy about that because I would be happy for the patients if they got added value from it and had more information and said: Hey, I didn't know that yet. And it's nice that I now have something at my fingertips. And when they're really happy, of course, I'm really happy too. Yes. (Phase2\Interview_02:104)

TDF = Theoretical Domains Framework; VR = Virtual Reality, VRPI = VR-based psycho-educational intervention
